# Floors for Hospital Wards

**Published:** 1895-06-08

**Authors:** 


### PRACTICAL DEPARTMENTS.

### FLOORS FOR HOSPITAL WARDS.

The construction of walls and floors is a matter of the first importance in all buildings which are to be used for the accommodation of sick people and the treatment of disease. Considerable difference of opinion will naturally be found, even amongst experts on the subject, as to what constitutes the best form of construction and material for ward floors, but there are certain broad principles which may be laid down as essential, and which should be understood by those who are responsible for the erection of new buildings if their adoption is to be ensured. It would seem almost needless to point out the necessity for a ward floor to be laid in such a manner as to render permeations of dust and organic matter well-nigh an impossibility; yet it is, unfortunately, only too possible to go into institutions, new institutions moreover, where gaping chinks may be discerned between the boards or blocks of the floor.

The first necessity for hospital flooring is that it shall be fire-proof or solid. This can be accomplished in various ways, the beams or joists being of iron or steel, embedded in concrete. In the New General Hospital, Birmingham, Mr. Henman, the architect, is proposing to use "adamant and breeze concrete blocks." Having noticed, he says, in a paper read before the Leeds and Yorkshire Architectural Society last year, "how unpleasantly noise is conveyed by so resonant a material as cement concrete," he "induced the manager of the Birmingham Adamant Company to carry out some experiments with a view to overcoming this objection," which it is believed can be done in this way: "The idea is based on the well-known fact that materials of different densities in juxtaposition more or less neutralise sound vibrations. It is believed that this method will be found particularly fire-resisting, because the joisting is thoroughly encased with a material which will resist heat, and is but slightly affected by the application of water after it has been exposed to a high temperature." The cost is also less than in other methods of fire-resisting construction.

With regard to the floor surface, oak or teak should be employed (the latter being the hardest, and preferred by many hospital authorities), if wood is the material to be used. Mr. Keith Young, whose experience in hospital construction is very considerable, advocates the use of "terrazzo," a mixture of Portland cement and marble chips, which is excellent for its non-absorbent qualities, and will take a fair polish. Terrazzo, "when well laid, and when care is taken to have a sufficient thickness of concrete over the iron joists, gives a solid and homogenous surface that is hard to beat." So says Mr. Young; and in Mr. Burdett's "Hospitals and Asylums" practically the same advice is given, at least, as to waiting-halls and offices. The objection which naturally occurs in considering its use for ward floors is that the substance is a cold and therefore not altogether a comfortable one for the feet, and it is just a question whether its safety and sanitary qualities outweigh this inconvenience. Terrazzo, or some similar material, has been, we believe, used throughout in the Derbyshire Royal Infirmary, and there meets with entire satisfaction. It is not found that the floor is noticeably colder than wood. It is cleansed with soap and water twice during the week. This treatment seems to diminish the polished surface, but once or so during the year swabbing with turpentine will, it is hoped, renew the surface. Another disadvantage is that these floors very easily show and seem to retain stains. There is a superior form of this flooring, known as "Mischiati," which Mr. Burdett especially recommends for use in the operation theatre, and in the mortuary and *post-mortem* room. There is in this a larger proportion of marble to cement, the surface being formed of cubes of marble laid close together, with no regular pattern. It takes a better polish and looks brighter.

(*To be continued.*)

